# First detection and impact of bovine herpesvirus type 4 on dairy cattle reproduction in Thailand

**DOI:** 10.14202/vetworld.2024.2259-2266

**Published:** 2024-10-07

**Authors:** Ruchikon Jongsuwanwattana, Piyathip Setthawong, Siriwat Suadsong, Sudson Sirivaidyapong, Theerawat Swangchan-Uthai

**Affiliations:** 1 Department of Obstetrics, Gynaecology and Reproduction, Faculty of Veterinary Science, Chulalongkorn University, Bangkok 10330, Thailand; 2 CU Animal Fertility Research Unit, Chulalongkorn University, Bangkok, Thailand; 3 Department of Physiology, Faculty of Veterinary Medicine, Kasetsart University, Bangkok 10900, Thailand

**Keywords:** bovine herpesvirus-4, dairy cattle, endometritis, phylogenetic analysis, reproductive performance, Thailand

## Abstract

**Background and Aim:**

Postpartum reproductive tract infections pose significant challenges to dairy farms, leading to economic losses due to reduced fertility associated with uterine inflammation. In veterinary practice, numerous research groups have explored the underlying causes of subfertility in cows, including surveying endemic viral infections related to endometritis in local areas. This study investigated bovine herpesvirus 4 (BoHV-4) infection in Thai dairy herds and assessed its impact on endometritis and subsequent reproductive outcomes.

**Materials and Methods:**

The present study analyzed BoHV-4 DNA in various samples, including milk, blood, and endometrial tissue, from 44 Holstein-Friesian cows 21–47 days postpartum across five dairy herds in Central Thailand. BoHV-4 glycoprotein B and thymidine kinase DNA sequences were detected using the polymerase chain reaction (PCR) and nested PCR, with sequence comparisons made to GenBank data for phylogenetic analysis. The endometritis status was diagnosed through vaginal mucus examination and endometrial cytology, with reproductive performance monitored up to the subsequent calving.

**Results:**

BoHV-4 DNA was identified in blood and endometrial tissues (15.91%) but not in milk samples. Phylogenetic analysis revealed that the local BoHV-4 strains are similar to those identified in Brazil and Japan. Notably, the presence of BoHV-4 was correlated with reduced postpartum reproductive performance, particularly extending the interval from calving to the first service.

**Conclusion:**

Our findings underscore the importance of integrating BoHV-4 genomic surveys and uterine health assessments to refine reproductive management strategies within the dairy industry.

## Introduction

The postpartum period is a critical phase that affects both cow fertility and the economic viability of dairy farms. Uterine bacterial contamination after calving is a natural occurrence in dairy cows and is typically resolved through uterine involution through mucosal barriers and innate immunity rather than acquired immunity. However, if not adequately controlled or eliminated, contamination can manifest as postpartum reproductive disorders, such as metritis and endometritis [1]. Uterine defense mechanisms in dairy cattle become compromised due to factors such as poor energy status and prior viral infections, particularly bovine herpesvirus 4 (BoHV-4) and bovine viral diarrhea virus, exacerbating the stressors inherent to this period. These compromised defense mechanisms significantly impede reproductive performance across dairy herds [2]. Both clinical and subclinical endometritis correlate with subfertility and infertility, resulting in prolonged calving to conception intervals, a higher number of services per conception, elevated culling rates, and increased treatment expenses [3, 4].

BoHV-4 is the primary viral infection frequently associated with uterine pathology initiated by the virus infection alone or concurrent bacterial infection. Despite being isolated worldwide from cows with pneumonia and various uterine diseases, i.e., vaginitis, metritis, and endometritis, BoHV-4 has also been detected in healthy cows, leading to debates regarding its pathogenic role in the endometrium [5, 6]. Although the association between BoHV-4 infection as a primary pathogen in uterine disease remains unclear, the virus has been identified as highly tropic for endometrial cells, subsequently causing a cytopathic effect [7–9], leading to endometrial tissue damage and inflammation [10]. Furthermore, BoHV-4 has been isolated from aborted fetuses, indicating its potential role in bovine reproductive failure [11].

Although the BoHV-4 virus has been isolated from various biological sources such as serum, milk, respiratory exudate, vaginal discharge, and placenta [12–14], its detection in genital isolates is particularly crucial for identifying cows at risk of endometritis. Polymerase chain reaction (PCR) and its variants, like nested PCR, are currently considered rapid and sensitive methods for detecting BoHV-4 DNA [3, 5], offering significant advantages in the surveillance of bovine viral diseases and the analysis of nucleotide sequencing [15]. Specifically, BoHV-4 glycoprotein B (gB) and thymidine kinase (TK) are the specific markers for identifying the virus within bovine specimens and tracing the demographic origins of the strains [16]. Understanding the underlying viral causes of bovine infertility, such as BoHV-4, is vital for informed decision-making in veterinary practice, ultimately enhancing reproductive management strategies in the dairy industry.

Therefore, this study aimed to detect the field isolates of BoHV-4 in dairy herds in Thailand using PCR and nested PCR assays, to perform a comprehensive phylogenetic analysis of the recovered strains and to assess the impact of BoHV-4-associated endometritis on the reproductive performance of affected cows.

## Materials and Methods

### Ethical approval

All animal handling and sampling procedures were conducted in accordance with the ethical standards of the Chulalongkorn University Animal Care and Use Committee No.1531012, Faculty of Veterinary Science, Chulalongkorn University, Thailand.

### Study period and location

The study was conducted from December 2021 to September 2023 on five commercial dairy farms in the provinces of Nakhon Pathom and Saraburi in Central Thailand.

### Experimental design

Postpartum Holstein-Friesian cows (n = 44) after 21–47 days postpartum were included in this experiment. Five bulk milk tanks, blood, and endometrial tissue samples were collected from five dairy herds to investigate BoHV-4 infection. The following DNA extraction, PCR, and nested PCR were performed to identify the presence of BoHV-4 gB and TK DNA, respectively. The retrieved amplicons were sequenced, aligned, and compared with existing BoHV-4 DNA sequence data from GenBank. The phylogenetics of the retrieved genomes were analyzed to elucidate the evolution of BoHV-4 and to compare field-test viral strains and other published sequences. In addition, the endometritis status in each experimental cow was determined by evaluating the characteristics of the vaginal mucus combined with endometrial cytology. The reproductive performance data of these cows were recorded over a period that extended to subsequent calving.

### Sample collection

Bulk tank milk, blood, and endometrial tissue samples were collected individually for DNA extraction. Within 1 day after collection, the milk samples were defatted by centrifuging at 4°C for 4 h, and the fraction below the lipid layer was collected for 1 mL. Blood samples were collected from the cows in heparinized blood collection tubes. The defatted milk and blood samples were stored at −20°C until DNA extraction [17]. Endometrial tissue samples were collected using the brush cytology technique [18], stored in 1 mL of Trizol reagent (Invitrogen, Waltham, MA, USA), and stored at 4°C until DNA extraction.

### DNA extraction

DNA was extracted from milk and blood samples by column chromatography using a DNeasy blood and tissue kit (Qiagen, Westburg, The Netherlands) following the manufacturer’s instructions. Before DNA extraction, 1000 μL defatted milk samples were centrifuged at 20,000× *g* for 3 min. A volume of 800 μL was discarded, and the cell pellet was resuspended in the remaining 200 μL. In contrast, 100 μL of each blood sample was adjusted to volume of 220 μL by adding phosphate-buffered saline. Milk and blood samples were digested with protease K, DNA precipitation, and followed by DNA elution. For endometrial samples, DNA was isolated using chloroform and subsequently precipitated using ethanol, following the guidelines provided by Life Technologies Corporation. DNA pellets were digested with protease K, precipitated, and washed using a DNeasy blood and tissue kit (Qiagen). The DNA extraction products were stored at –70°C.

### gB and TK PCR assays

For the amplification of BoHV-4 gB DNA templates (615 bp), PCR was performed as described by Wellenberg *et al*. [19] using PCR Go Taq® Green Mastermix (Promega, Madison, WI, USA): the forward primer gB1 (5-CCCTTCTTTACCA CCACCTACA-3) and the reverse primer gB2 (5-TGCCATAGCAGAG AAACAATGA-3). The program started with one step at 95°C for 10 min, followed by 45 cycles of 94°C for 60 s, 58°C for 60 s, and 72°C for 90 s. The amplification was completed by an ultimate elongation step at 72°C for 7 min.

The amplification of BoHV-4 TK DNA templates (215 bp) was performed using a modified nested TK-PCR approach as described by Egyed *et al*. [6]. Brief modifications were made to the PCR Mastermix components: Specifically, DNA templates were amplified using PCR GoTaq® Green Mastermix (Promega, Madison, USA). These primer sequences were used in this study: TK1: 5-GTTGGGCGT CCTGTATGGTAGC-3,TK2: 5-ATGTATGCCCAAAACTTATAATATGAC CAG-3, TK3: 5-TTGATAGTGCGTTGTTGGGATGTGG-3, and TK4: 5-CACTGCCCGGTGGGAAATAGCA-3. In both the initial and nested PCR rounds, the following thermal cycling program was employed to amplify BoHV-4 TK templates: initial denaturation at 95°C for 10 min, followed by 30 cycles consisting of 94°C for 60 s, 60°C for 60 s, and 72°C for 90 s. Subsequently, 5 μL of the PCR product from the first PCR round was subjected to further amplification in a nested PCR round using the same PCR mixture with the nested primers 3 and 4. The amplification was finalized by an ultimate elongation step at 72°C for 7 min. Amplicons were visualized by electrophoresis (Bio-Rad Laboratories, Hercules, California, USA) on 1% agarose gels. The gel images were captured using an ultraviolet illuminator (Bio-Rad Laboratories).

### Sequence and phylogenetic analysis

Nucleotide sequences of the purified PCR products of BoHV-4 gB and BoHV-4 TK DNA from this study were determined using an Applied Biosystems genetic analyzer with the BigDye® Terminator v3.1 cycle sequencing kit (First BASE Laboratories, Selangor, Malaysia). The homology between the recovered and published BoHV-4 DNA sequences is observed with the BLAST program (Basic Local Alignment Search Tool, http://blast.ncbi.nlm.nih.gov/Blast.cgi).

Assembled consensus sequences of the isolates and reference BoHV-4 sequences (gB DNA: GenBank ID ON156607.1, AJ609274.1, JN133502.1, KC540702.1, MH799319.1, AF318573.1, Z15044.1, OP631674.1, MN735176.1, MG181946.1, MG264405.1, KU180396.1, KP209031.1 MK543551.1, GQ375280.1, MK095406.1, MN551084.1 and KC999113.1, and TK DNA: GenBank ID OP631674.1, MZ463068.1, MN551084.1, MN735172.1, MK095375.1, MN173774.1, MG242032.1, KT166423.1, KC999113.1, KP209015.1, JX644990.1, JQ838046.1, JN133502.1, EU244697.1, AF318573.1, AB035517.1 and S49773.1) were edited and analyzed using the Molecular Evolutionary Genetics Analysis 11 (MEGA11) software package [20]. The sequence alignments were performed using ClustalW tool [21], which is included in MEGA 11 software. Model selection for the phylogenetic analysis was performed using the Bayesian Information Criterion based on nucleotide alignment. Evolutionary analysis and phylogenetic reconstruction were conducted [22]. The evolutionary history was inferred using the maximum-likelihood method based on model selection [23], and the bootstrap values were calculated using 1000 replicates. The tree with the highest log likelihood is shown. The percentage of trees with clustered associated taxa is shown next to the branches. Initial trees for the heuristic search were obtained automatically by applying the neighbor-join and BioNJ algorithms to a matrix of pairwise distances estimated using the Maximum Composite Likelihood approach and then selecting the topology with the superior log likelihood value. The tree was drawn to scale, and branch lengths were measured to determine the number of substitutions per site. All positions with missing data were eliminated. Finally, evolutionary analyses were conducted.

### Endometritis assessment

In each cow, endometritis was diagnosed based on a standard protocol described in a previous report [24]. Vaginal mucous samples were collected using a Metricheck™ device (Simcro, Hamilton, New Zealand) for endometritis assessment and characterized based on mucous consistency. Clinical endometritis was defined as the presence of a purulent material exceeding 50% or mucopurulent discharge containing approximately 50% pus in the vagina after 21 days postpartum. Subsequently, endometrial tissues were collected using the guard-swab endometrial brush cytology technique, smeared onto a glass slide, fixed with methanol, and stained with Diff Quick^®^ stain (Clinag Co., Ltd., Bangkok, Thailand). The cytological analysis involved determining the percentage of polymorphonuclear neutrophils (PMNs) by counting at least 100 cells at 400× magnification. Cows were diagnosed with subclinical endometritis if more than 18% PMNs were present at 20–33 days postpartum or more than 10% PMNs at 34–47 days postpartum.

### Statistical analysis

Statistical analyses were conducted using IBM SPSS for Windows, Version 28.0 (IBM Corp., NY, USA). Reproductive performance data were collected, including the number of services per conception, intervals from calving to first heat, calving to first service, first heat to first service, calving to conception, first service to conception, and overall calving interval. These metrics were used to analyze the association between BoHV-4 and endometritis. Descriptive statistics for reproductive performance are presented as mean ± standard error of the mean. The relationship between BoHV-4 infection and endometritis was assessed using the Pearson Chi-square test. The statistical models accounted for the fixed effects of BoHV-4, endometritis, and their interaction, with reproductive indices serving as independent variables. These data were analyzed using a multiple analysis of variance through the general linear model procedure. p < 0.05 was considered statistically significant.

## Results

### Expression of BoHV-4 gB and TK DNA using PCR

PCR targeting BoHV-4 gB DNA was applied to six samples, revealing the presence of the virus in 33.33% (2/6) of endometrial tissue samples and 16.67% (1/6) of blood samples. Meanwhile, a nested PCR assay aimed at detecting BoHV-4 TK DNA was conducted on 38 endometrial tissue samples. Of these, 11.76% (4/38) showed positive results. Across all samples tested, including endometrial tissues and blood, BoHV-4 DNA was detected in 15.91% (7/44) of cases. No BoHV-4 DNA was detected in any of the bulk tank milk samples collected from the five dairy herds.

### Nucleotide sequence and phylogenetic analysis

PCR products were sequenced to validate the identity of BoHV-4 DNA. Using nucleotide BLAST, we observed high homology between our field isolates and published strains. BoHV-4 TK DNA showed the highest homology (96.35%) with the BoHV-4 long unique region (accession number NC_002665.1), followed by BoHV-4 gB DNA (94.10%) compared with the same reference sequence.

Further analysis involved 18 referenced gB gene sequences and sixteen TK gene sequences from GenBank to determine the relatedness of our field strains to previously published strains. Phylogenetic trees for the gB and TK genes were constructed using the maximum-likelihood method, employing the Hasegawa–Kishono–Yano model and the Tamura 3-parameter (T92) model, respectively, with bootstrap replication values of 1000.

**Figure-1:**
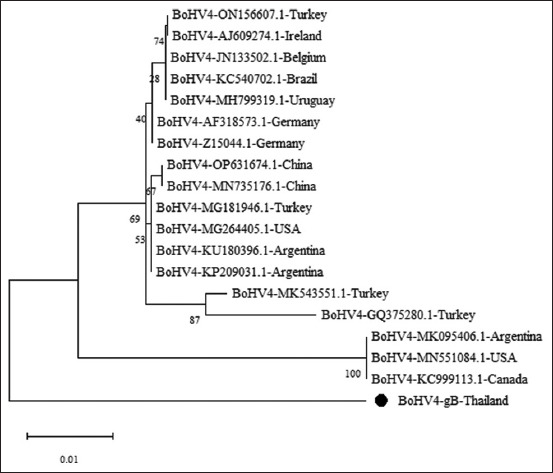
Molecular phylogenetic analysis of BoHV-4 gB gene nucleotide sequences using the maximum likelihood method. This phylogenetic tree compares the nucleotide sequences of our study with 18 known BoHV-4 strains from various geographic locations. The field strain identified in this study is indicated by a black circle. BoHV-4=Bovine herpesvirus 4, gB=Glycoprotein B.

The phylogenetic tree for BoHV-4 gB DNA (Figure-1) was divided into three main clades: Clade 1 contained three lineages, with Lineage 1 comprising strains from Belgium, Brazil, China, Germany, Ireland, Turkey, Uruguay, and the USA. Lineage 2 included strains from Argentina, China, Turkey, and the USA, whereas lineage 3 consisted of a single strain from Turkey. Clade 2 included strains from Argentina, Canada, and the USA. Clade 3 exclusively included field strains from this study, positioned in a distinct branch, illustrating geographic differentiation.

**Figure-2:**
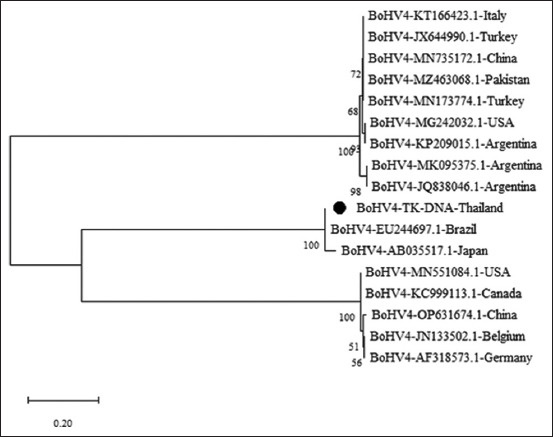
Molecular phylogenetic analysis of BoHV-4 TK gene nucleotide sequences using the Maximum Likelihood Method. The phylogenetic tree incorporates the nucleotide sequences identified in this study along with those of 16 known BoHV-4 strains from diverse geographic regions. The field strain identified in this study is indicated by a black circle. BoHV-4=Bovine herpesvirus 4. TK=Thymidine kinase.

Similarly, Figure-2 presents the phylogenetic analysis of BoHV-4 TK DNA, which was also segmented into three clades: Clade 1, split into two lineages featuring strains from Argentina, China, Italy, Pakistan, Turkey, and the USA; Lineage 2 specifically included strains from Argentina. Clade 2 comprised strains from Brazil and Japan, with our Thai field isolates showing similarities, indicating a potential shared lineage. Clade 3 included strains from Belgium, Canada, China, Germany, and the USA, highlighting the diversity and widespread nature of BoHV-4.

### Endometritis assessment

A comprehensive dataset on postpartum endometritis status and reproductive performance was collected from a cohort of 44 cows. Among these, seven cows tested positive for BoHV-4. Within the endometritis group, 65.91% (29/44) were diagnosed with either clinical or subclinical endometritis, and of these, six were positive for BoHV-4 DNA in their endometrial tissue samples. In contrast, the control group comprised 34.09% of the cohort (15/44 cows), with only one cow testing positive for BoHV-4 DNA through PCR detection (Table-1). The statistical analysis revealed no significant association between endometritis and BoHV-4 infection. In the subgroup analysis, however, tissue from endometritis was 3.7 times more likely to contain BoHV-4 than endometrial tissue from control individuals.

**Table-1:** Frequency table of cases of bovine herpesvirus–4-associated endometritis in dairy cows.

BoHV-4 status	Endometritis positive (%)	Endometritis negative (%)
BoHV-4 positive	6 (13.64)	1 (2.72)
BoHV-4 negative	23 (52.27)	14 (31.82)

BoHV-4=Bovine herpesvirus 4

### Effects of BoHV-4 infection and endometritis on reproductive performance

Reproductive performance data for all cows in this study were collected from the time of sample collection until the subsequent parturition. Three cows that tested positive for BoHV-4 were excluded from this specific analysis because they were culled before exhibiting the first signs of estrus post-calving.

The effects of BoHV-4 infection and endometritis on reproductive performance in 41 postpartum cows are presented in Table-2. As expected, the intervals from calving to the first heat and from calving to the first service were significantly prolonged in the endometritis group, with averages of 71.77 ± 7.42 day and 95.04 ± 5.40 day, respectively. In contrast, the nonendometritis group, which averages 45.47 ± 4.51 day (p = 0.008) and 73.33 ± 3.73 day (p = 0.004), respectively. There were no significant differences in the number of services, the interval from the first heat to the first service, calving to conception, first service to conception, or the overall calving interval between the endometritis and non-endometritis groups (p > 0.05).

**Table-2:** Reproductive performance of 41 postpartum dairy cows in this study: Comparison between groups with and without BoHV-4 infection and endometritis status (Mean ± SEM).

Reproductive indices	BoHV-4		Endometritis	
	
	Positive (n = 4)	Negative (n = 37)	Positive (n = 26)	Negative (n = 15)
Number of services per conception (times)	3.00 ± 0.58	3.27 ± 0.30	3.35 ± 0.36	3.07 ± 0.43
Calving to the first heat interval (day)	72.75 ± 24.42	61.00 ± 5.41	71.77 ± 7.42^a^	45.47 ± 4.51^b^
Calving to the first service interval (day)	109.00 ± 25.02^a^	84.73 ± 3.54^b^	95.04 ± 5.40^a^	73.33 ± 3.73^b^
First heat to first service interval (day)	36.25 ± 24.56	23.73 ± 3.91	23.27 ± 5.54	27.87 ± 6.17
Calving to conception interval (day)	220.00 ± 55.37	264.78 ± 33.47	263.46 ± 31.62	255.13 ± 64.95
First service to conception interval (day)	111.00 ± 39.42	180.05 ± 33.03	168.42 ± 29.93	181.80 ± 65.63
Calving interval (day)	491.50 ± 54.31	541.59 ± 34.15	539.27 ± 32.68	532.27 ± 65.58

^a,b^Within a row, means without a common superscript differ (p < 0.05). SEM=Standard error of the mean, BoHV-4=Bovine herpesvirus 4

Regarding BoHV-4 infection, calving to the first service interval was significantly longer for cows that tested positive (109.00 ± 25.02 day) compared to those testing negative (84.73 ± 3.54 day) (p = 0.036). However, similar to the analysis of the impact of endometritis, no significant differences were found in the number of services, intervals from calving to first heat, from first heat to first service, from calving to conception, from first service to conception, and in the calving interval between the BoHV-4-positive and BoHV-4-negative groups (p > 0.05).

## Discussion

The present study is the first reported isolation of BoHV-4 from the endometrium of dairy cows in Thailand, coupled with genetic characterization and analysis of reproductive performance throughout the calving cycle. Consistent with global findings, our study identified BoHV-4 as a significant endemic disease within Thai dairy herds, with a prevalence of 15.91%, closely aligning with the global range of 16%–33% [10–12, 20]. Consequently, we propose that identifying BoHV-4 in the endometrium of postpartum cows may elucidate the virus’s role in precipitating endometritis, potentially contributing to subfertility and reduced reproductive performance in affected cows.

Consistent with previous investigations on BoHV-4 detection in dairy herds [25, 26], this study utilized gB PCR and nested TK PCR methods based on their proven specificity, sensitivity, reproducibility, and rapidity in detecting BoHV-4 DNA within bovine specimens. These techniques enhanced our ability to efficiently screen various sources of bovine samples, allowing for a more precise assessment of the prevalence of BoHV-4 infection. Notably, BoHV-4 gB DNA, an essential component for viral infectivity, is conserved across all BoHV-4 strains [27]. Despite its known tropism in mammary tissues [28], our study did not detect it in bulk tank milk. Instead, its presence in blood and endometrial tissues suggests active virus replication in the reproductive tract, transient viremia, and latent infections in lymphocytes and endometrial cells [26, 29]. Our study extended this analysis to endometrial tissue using nested PCR for BoHV-4 TK DNA, which exhibits greater intensity. This finding underscores the importance of nested PCR for detecting viral DNA in cattle tissues and highlights the utility of the TK gene as a crucial marker for distinguishing between vaccinated and naturally infected animals [7, 8]. Moreover, using these specific BoHV-4 amplicons enables tracing the demographic origins of strains [12], thereby aiding in elucidating the genetic lineage of BoHV-4 in Thai dairy herds through phylogenetic analysis.

There is limited research on the demographic impacts of BoHV-4 in dairy cattle. Our study found that BoHV-4 gB DNA from blood samples from Thailand forms a distinct branch, suggesting that strains from other regions do not share evolutionary lineages. Conversely, the Thai BoHV-4 TK strains shared a lineage with strains from Brazil and Japan, suggesting extensive virus dissemination through international trade. Current international protocols for screening donor sires and dam do not include BoHV-4, despite its presence in reproductive tissues [30, 31] and fluids [32]. It is important to integrate BoHV-4 screening into future Assisted Reproductive Technologies (ARTs) to prevent international transmission. Additionally, the World Organization for Animal Health 2023 classified BoHV-4 as a Category 4 disease [33], i.e., pathogenic agents under study that either lack conclusive data regarding transmission risk or present a non-negligible risk of transmission via embryo transfer. This emphasizes the need to update biosecurity measures for ARTs to ensure the safety and health of livestock on a global scale.

After calving, the endometrium is exposed to environmental bacteria, which often leads to endometritis when uterine defense mechanisms fail to counteract the infection [34]. Specimens for this study were collected from dairy cows 21–47 days postpartum, a critical period marked by significant stress that can influence BoHV-4 dynamics [35]. Preceding viral infections, including BoHV-4, have been implicated in compromising reproductive performance and inducing infertility. Epidemiological data indicated a higher seroprevalence of BoHV-4 in cows with metritis, abortion, and infertility compared to healthy cows, suggesting its potential for identifying reproductive disorders [36]. However, not all studies have confirmed this link [25, 37]. Recent genomic analyses have identified BoHV-4 as a potential reproductive pathogen [13, 38], which often exacerbates uterine inflammation in combination with bacterial infections [27, 39]. In this context, endometrial tissue samples from cows with endometritis were more likely to contain BoHV-4 (odds ratio = 3.7). However, this difference did not reach statistical significance (confidence interval = 0.40–33.59), possibly due to small sample sizes [40] or confounding factors like stress [2] or the presence of specific pathogens, particularly endometrial pathogenic *Escherichia coli* [41]. This finding supports the theory that infection of the cow uterus paves the way for subsequent viral infection [24].

The investigation of BoHV-4’s impact on bovine fertility is still emerging, with methodological differences complicating comparisons across studies. Similar to a previous report that BoHV-4 infection reduced the chance for insemination within 80 days postpartum [42], our findings confirmed that BoHV-4 and endometritis significantly prolonged calving to the first service interval, with the worst outcomes observed in cows affected by both conditions, extending the interval up to an average of 130 days compared with having either BoHV-4 or endometritis alone at 109 days and 95 days, respectively. Additionally, postpartum uterine infections can delay the endometrial regeneration and resumption of ovarian activities, thereby delaying the first insemination. Notably, cows with endometritis frequently lack palpable ovarian structures and exhibit reduced estrus detection and conception rates, leading to extended reproductive intervals [43]. Our findings underscore the negative impact of BoHV-4 alone and concurrent bacterial infections, leading to endometritis and subsequent reproductive failure [44], including higher rates of repeat breeding, reduced pregnancy rates, increased services per conception, prolonged calving to conception intervals, and higher abortion rates [45–47]. These findings highlight the complex interaction between infectious agents and reproductive health in dairy cattle, underscoring the need for comprehensive management strategies to mitigate these impacts and enhance overall herd fertility.

## Conclusion

The present study emphasizes the importance of conducting genomic surveys on BoHV-4, targeting affected sites, such as the endometrium, and analyzing its association with uterine pathology via a comprehensive case/control approach. These measures are crucial for enhancing reproductive management practices in the dairy sector. To our knowledge, this study is the first to document the prevalence of BoHV-4 and its impact on the reproductive performance of dairy cattle in Thailand. Using PCR assays, we detected BoHV-4 gB and TK DNA fragments directly from endometrial tissues and whole blood samples. Our findings revealed that BoHV-4 TK DNA from Thai strains shares evolutionary connections with strains from East Asia and South America, suggesting that these strains are likely to have international transmission. Further investigations are warranted to clarify the specific roles of different BoHV-4 genotypes in the pathogenesis of uterine infections and subsequent inflammation. These studies will provide valuable insights for the veterinary profession and dairy farmers, thereby enhancing overall herd health and productivity.

## Authors’ Contributions

RJ: Data acquisition and analysis and drafted the manuscript. PS: Interpretation of the data and reviewed the manuscript. SSu: Data acquisition. SS: Conception of the study and reviewed the manuscript. TS: Conception of the study, data acquisition and analysis, and integrity of all aspects of the study. All authors have read and approved the final manuscript.
